# Breaking It Down: A Systematic Review Unravelling the Impact of Attention Deficit Hyperactivity Disorder and Methylphenidate on Childhood Fractures

**DOI:** 10.7759/cureus.56833

**Published:** 2024-03-24

**Authors:** Gourav Garg, Lotanna Umeano, Sadaf Iftikhar, Sarah F Alhaddad, Christian N Paulsingh, Muhammad Faisal Riaz, Safeera Khan

**Affiliations:** 1 Orthopaedics, King's Mill Hospital, Sutton-in-Ashfield, GBR; 2 Internal Medicine, Neurology, California Institute of Behavioral Neurosciences & Psychology, Fairfield, USA; 3 Internal Medicine, California Institute of Behavioral Neurosciences & Psychology, Fairfield, USA; 4 Pediatric, California Institute of Behavioral Neurosciences & Psychology, Fairfield, USA; 5 Pathology, St. George's University School of Medicine, St. Georges, GRD; 6 Internal Medicine, Rawalpindi Medical University, Rawalpindi, PAK; 7 Neuropsychiatry, California Institute of Behavioral Neurosciences & Psychology, Fairfield, USA

**Keywords:** traumatic injury, extremity fractures, pediatric fractures, stimulant treatment, attention-deficit/ hyperactivity disorder, trauma and orthopedics, fracture in a child, methylphenidate (mph), fracture, attention deficit hyperactivity disorder (a.d.h.d.)

## Abstract

Limb fractures are a common cause of pediatric hospital admissions and surgeries, with a significant prevalence in the United Kingdom across all injury categories. Among psychiatric conditions in children, attention deficit hyperactivity disorder (ADHD) stands out as frequently associated with fractures, particularly those involving extremities. ADHD, with diagnoses prevalent among a significant proportion of school-age children and adolescents, has witnessed a growing global incidence. We followed the Preferred Reporting Items for Systematic Reviews and Meta-Analyses (PRISMA) 2020 checklist for our systematic literature search, using various databases and specific search terms related to ADHD and fractures. We considered articles from 2018 to 2023, focusing on English language papers with free full-text access. Our selection process used the PRISMA flowchart. We began with 1,890 articles and, after deduplication, title screening, abstract assessment, and quality evaluation included nine research papers in our review. Our primary focus was on examining fracture-related outcomes in individuals with ADHD compared to those without, considering medication status. These studies encompassed various designs, with a focus on the ADHD-fracture relationship and methylphenidate’s (MPH) impact. Our study confirms that ADHD increases fracture risk and suggests that MPH may help mitigate this risk. Early ADHD detection is vital for nonpharmacological interventions. Orthopedic surgeons should proactively identify ADHD, while healthcare professionals should offer injury prevention guidance, particularly for at-risk groups.

## Introduction and background

Limb fractures are one of the most common causes of pediatric hospital admissions and surgeries [1]. Fractures are one of the most frequent types of injury in the United Kingdom, even when all other categories are taken into account [2]. If we consider all psychiatric conditions in children, then the most common condition associated with fractures is attention deficit hyperactivity disorder (ADHD) [3,4]. More specifically, it is also the most commonly studied psychiatric condition associated with extremity fractures in children [5,6].

ADHD is a neurodevelopmental disorder diagnosed in around 3-5% of school-age children and adolescents [7]. Additionally, children and adolescents with ADHD have also been reported to experience certain coexisting emotional conditions like anxiety and depression [8]. The worldwide occurrence of ADHD has displayed a consistent rise over the last few decades [9]. A meta-analysis released in 2015 determined an overall prevalence rate of 7.2% [10]. More current statistics indicate that 9.4% of children and adolescents in the United States have received an ADHD diagnosis at some point [11,12].

ADHD is categorized into three primary subtypes: hyperactivity-impulsivity ADHD, inattention ADHD, and combined inattentive/hyperactive-impulsive ADHD, often referred to as combined ADHD [13-16].

The diagnosis of ADHD primarily relies on gathering information from the child’s parents, school, and, if consulted, healthcare professionals. This process is complemented by interviews and examinations [16-18]. Numerous scales and questionnaires are accessible for evaluating ADHD characteristics in an individual or for aiding in the diagnostic process.

Being diagnosed with ADHD has been demonstrated to elevate the likelihood of experiencing a traumatic fracture. This correlation is likely attributable to the behavioral traits linked to the disorder, which encompass recklessness, clumsiness, an elevated inclination to disregard rules in games or sports, and a diminished focus on safety precautions [19,20]. Most studies suggest that all fracture outcomes are more common in children diagnosed with ADHD when compared to non-ADHD cohorts [12,16,21,22].

There are various treatment options available for children diagnosed with ADHD, including behavioral therapy and pharmacological treatment. Among pharmacological treatments, different classes of medications are available, broadly classified as stimulants and nonstimulants. Among stimulants, the phenidate group with the drug methylphenidate (MPH) is one of the most common medications used for ADHD. These medications are called stimulants because they stimulate specific parts of the brain, especially those that play a role in controlling attention and behavior [23].

The majority of research in the literature has focused on investigating the association between ADHD and fractures. Nevertheless, there remains a scarcity of comprehensive data regarding the intricacies of this connection. For instance, there is a need for a deeper understanding of how ADHD contributes to an elevated risk of fractures and the various factors that play a role in this relationship. Similarly, there is a need to delve into the relationship between MPH, including its mechanism of action and its overall impact on this association.

In this systematic review, our objective is to thoroughly investigate the connection between ADHD and fractures. We aim to examine various facets of ADHD, the contributing factors, and their impact on the occurrence and frequency of fractures in children and young individuals. Additionally, we will analyze the influence of MPH on the trajectory of fractures in cohorts diagnosed with ADHD. This comprehensive study aims to enhance our comprehension of this relationship, ultimately leading to improved strategies for preventing fractures in children. This, in turn, can alleviate the strain on countless families and the healthcare system.

## Review

Materials and methods

We used the Preferred Reporting Items for Systematic Reviews and Meta-Analyses (PRISMA) 2020 checklist to search for the relevant literature [24].

Search Sources and Strategy

We searched PubMed, PubMed Central (PMC), Medline, Google Scholar, and MDPI to search for the relevant literature. We used various combinations of ADHD, drug therapy, MPH, and fractures to search all the databases. However, in PubMed, adjacent to these keywords, the following strategy was formed and used to search relevant literature in PubMed’s MeSH (Medical Subject Headings) database: ("Attention Deficit Disorder with Hyperactivity/complications''[Mesh] OR "Attention Deficit Disorder with Hyperactivity/drug therapy"[Mesh] OR "Attention Deficit Disorder with Hyperactivity/therapy"[Mesh] ) AND "Methylphenidate/therapeutic use"[Majr] AND ("Fractures, Bone/epidemiology"[Mesh] OR "Fractures, Bone/prevention and control"[Mesh] ).

Table 1 below mentions all the sources, strategies used, and the number of papers identified in each of them.

**Table 1:** Search strategy, database, and number of papers identified

Search strategy	Database used	Number of papers identified
("Attention Deficit Disorder with Hyperactivity/complications"[Mesh] OR "Attention Deficit Disorder with Hyperactivity/drug therapy"[Mesh] OR "Attention Deficit Disorder with Hyperactivity/therapy"[Mesh] ) AND "Methylphenidate/therapeutic use"[Majr] AND ( "Fractures, Bone/epidemiology"[Mesh] OR "Fractures, Bone/prevention and control"[Mesh])	PubMed Mesh	4
((((attention deficit hyperactivity disorder[Title]) OR (ADHD[Title])) OR (attention deficit disorder with hyperactivity[Title])) AND (methylphenidate[Text Word])) AND (fracture[Text Word])	PubMed Advanced	2
((((attention deficit hyperactivity disorder[Title]) OR (ADHD[Title])) OR (attention deficit disorder with hyperactivity[Title])) OR (methylphenidate[Text Word])) AND (fracture[Text Word])	PubMed Advanced	15
Adhd AND fracture	PubMed	27
Methylphenidate AND fracture	PubMed	13
Adhd and fracture (("attention deficit disorder with hyperactivity"[MeSH Terms] OR ("attention"[All Fields] AND "deficit"[All Fields] AND "disorder"[All Fields] AND "hyperactivity"[All Fields]) OR "attention deficit disorder with hyperactivity"[All Fields] OR "adhd"[All Fields]) AND ("fractures, bone"[MeSH Terms] OR ("fractures"[All Fields] AND "bone"[All Fields]) OR "bone fractures"[All Fields] OR "fracture"[All Fields])) AND ("open access"[filter] AND ("2018/01/01"[PubDate] : "2023/12/31"[PubDate]))	PMC/Medline	977
(("attention deficit disorder with hyperactivity"[MeSH Terms] OR ("attention"[All Fields] AND "deficit"[All Fields] AND "disorder"[All Fields] AND "hyperactivity"[All Fields]) OR "attention deficit disorder with hyperactivity"[All Fields] OR "adhd"[All Fields]) AND ("fractures, bone"[MeSH Terms] OR ("fractures"[All Fields] AND "bone"[All Fields]) OR "bone fractures"[All Fields] OR "fracture"[All Fields]) AND ("methylphenidate"[MeSH Terms] OR "methylphenidate"[All Fields])) AND ("open access"[filter] AND ("2018/01/01"[PubDate] : "2023/12/31"[PubDate]))	PMC/Medline	105
Adhd AND methylphenidate AND fracture	Google Scholar	666
Adhd AND fracture AND methylphenidate	MDPI	9
Adhd AND fracture	MDPI	72
Total number of papers identified		1,890
Number of articles after duplicate removal (276)		1,614

Inclusion criteria: We identified the latest literature for the topic and, during the search, met the following inclusion criteria. Articles from 2018-2023, written in the English language, or if a full-text translation in English was available, were included. Articles comprising human participants of all genders were included. Articles with free full text online were selected. A few of the articles, which passed the screening process but whose full text was not freely available, were requested at the library at King’s Mill Hospital, Sherwood Forest Hospitals NHS Foundation Trust, Sutton-in-Ashfield, United Kingdom. Hence, access was provided by the library.

Exclusion criteria: Other articles for whom the full text could not be retrieved were excluded. Articles focusing on stress fractures and the relationship between ADHD and traumatic brain injuries, specifically those involving patients with rare comorbid conditions, were not included. Articles that included gross injuries without clearly mentioning whether fractures were included or not were also excluded. Gray literature and proposal papers were also rejected.

Selection Process

All the identified articles from all the databases were transferred to EndNote. Then, initially, duplicates were removed. Each article was screened by going through titles and abstracts and independently assessed by two authors. If there were any conflicts, those were discussed among the other co-authors, and hence the final decision was made by mutual consensus. Articles shortlisted after that were evaluated by going through the full text, and relevant ones were selected. All articles went through the inclusion and exclusion criteria and were shortlisted.

Quality Assessment of the Studies

All the shortlisted articles passed through the quality assessment tools. All co-authors did many quality checks. Observational studies were assessed on the JBI Critical Appraisal Checklist, while the Assessment of Multiple Systematic Review (AMSTAR) tool was used for systematic reviews. Only the studies that had satisfactory quality appraisals were included.

Data Collection Process

After the quality assessment, a final number of articles that were included in the systematic review were obtained. Then, the authors went through these articles multiple times to extract the data to be used for the study. Data was extracted both manually and via a data questionnaire method.

Results

Study Identification and Selection

**Figure 1:**
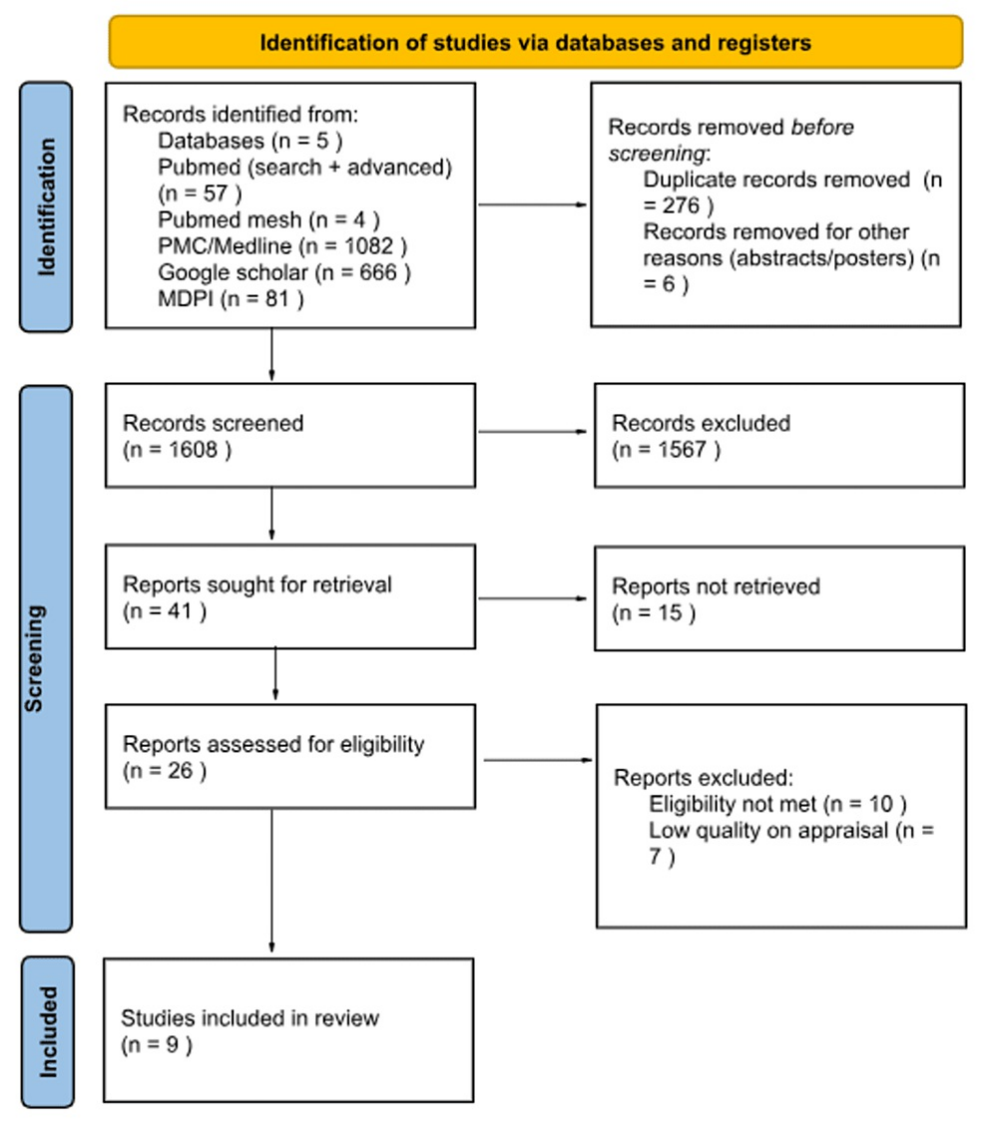
PRISMA flowchart depicting article selection PRISMA, Preferred Reporting Items for Systematic Reviews and Meta-Analyses

We initially located 1,890 articles across all the databases. After eliminating 276 duplicate articles, we subjected 1,608 remaining articles to title screening, of which only 71 met the criteria. Further assessment of the abstracts narrowed it down to 37 articles. Subsequently, we retrieved the full-text versions of these articles and conducted a thorough quality assessment. Ultimately, we included nine articles in our review. The detailed study selection process is visually represented in Figure 1 within the PRISMA flowchart.

**Figure 2:**
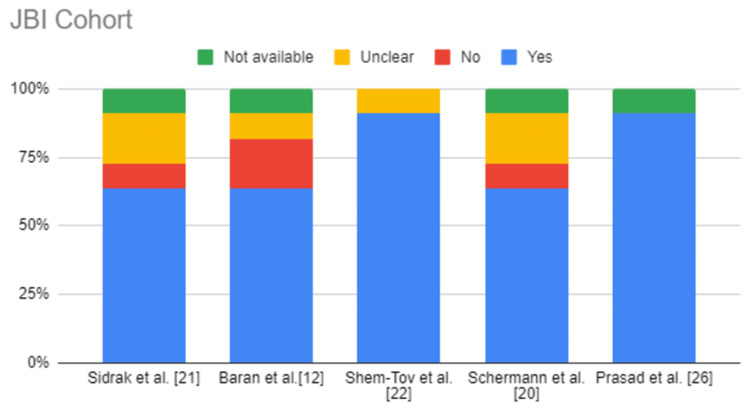
JBI Critical Appraisal Chart for cohort studies

**Figure 3:**
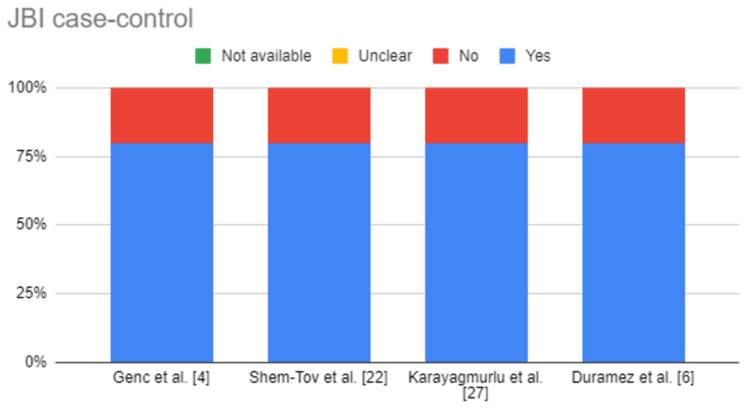
JBI Critical Appraisal Chart for case-control studies

**Figure 4:**
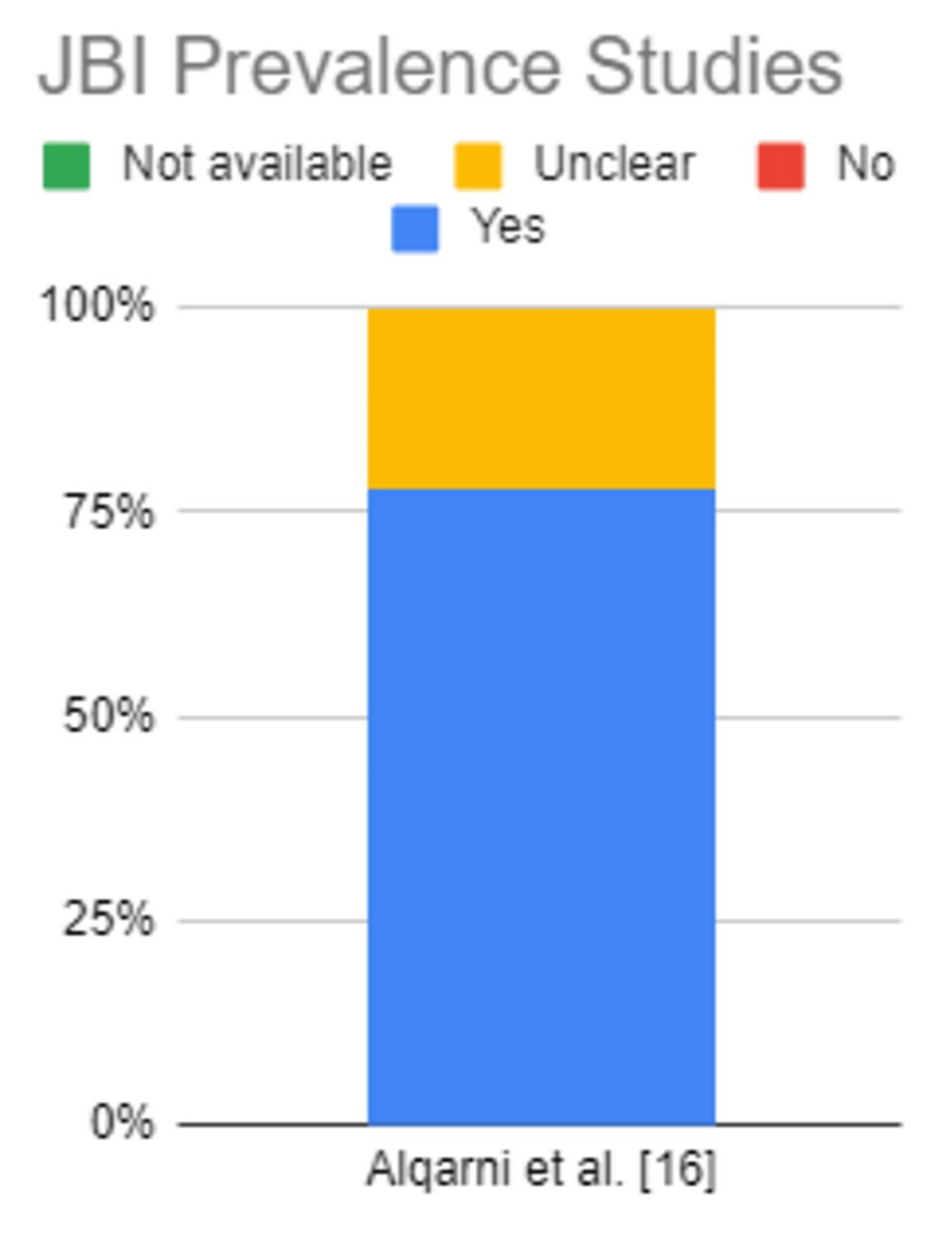
JBI Critical Appraisal Chart for prevalence studies

The articles were assessed for eligibility using the relevant quality appraisal tools [25]. Table 2, Table 3, and Table 4 show the results of the quality appraisal. Figure 2, Figure 3, and Figure 4 also depict the results of quality appraisal in chart format.

**Table 2:** JBI Critical Appraisal Tool for cohort studies

JBI cohort	Sidrak et al. [21]	Ziv-Baran et al. [12]	Shem-Tov et al. [22]	Schermann et al. [20]	Prasad et al. [26]
Similar groups from a similar population	Yes	Yes	Yes	Yes	Yes
Groups assigned after similar exposure measurements	Yes	Yes	Yes	Yes	Yes
A valid and reliable way of exposure measurement	Yes	Yes	Yes	Yes	Yes
Confounding factors identified	Unclear	No	Yes	No	Yes
Strategies to deal with confounding factors started	No	No	Yes	Unclear	Yes
Participants free of exposure at the start	Unclear	Unclear	Unclear	Unclear	Yes
Valid and reliable way of outcome measurement	Yes	Yes	Yes	Yes	Yes
Follow-up time reported or long enough for outcomes to occur	Yes	Yes	Yes	Yes	Yes
Follow-up complete or if not, then reasons are explained	Yes	Yes	Yes	Yes	Yes
Strategies to deal with incomplete follow-up	Not available	Not available	Yes	Not available	Not available
Appropriate statistical analysis	Yes	Yes	Yes	Yes	Yes
Overall appraisal	Include	Include	Include	Include	Include

**Table 3:** JBI Critical Appraisal Tool for case-control studies

JBI Case-Control	Genç et al. [4]	Shem-Tov et al. [22]	Karayagmurlu et al. [27]	Duramaz et al. [6]
Comparable groups (except disease)	Yes	Yes	Yes	Yes
Appropriate matching of cases and controls	Yes	Yes	Yes	Yes
The same criteria used for identification	Yes	Yes	Yes	Yes
A valid and reliable way of exposure measurement	Yes	Yes	Yes	Yes
The same way of exposure measurement	Yes	Yes	Yes	Yes
Confounding factors identified	No	No	No	No
Strategies to deal with confounding factors started	No	No	No	No
Valid and reliable way of outcome measurement	Yes	Yes	Yes	Yes
Exposure period of interest long enough to be meaningful	Yes	Yes	Yes	Yes
Appropriate statistical analysis	Yes	Yes	Yes	Yes
Overall appraisal	Include	Include	Include	Include

**Table 4:** JBI Critical Appraisal Tool for prevalence studies

JBI Prevalence Studies	Alqarni et al. [16]
Appropriate sample frame to address the target population	Yes
Appropriate way of sampling study participants	Yes
Adequate sample size	Yes
Study subjects and the setting described in detail	Yes
Data analysis was conducted with sufficient coverage of the identified sample	Yes
Valid methods used for the identification of the condition	Unclear
Condition measured in a standard, reliable way	Yes
Appropriate statistical analysis	Yes
Response rate adequate, and if not, was the low response rate managed appropriately	Unclear
Overall appraisal	Include

Outcomes Measured

The main focus of our analysis involved extracting primary outcomes from the selected research papers. These outcomes included examining fracture-related results in individuals with ADHD compared to those without ADHD in cohort studies. Additionally, we assessed individuals with ADHD who were on medication and compared them to those without ADHD who were not on medication, providing valuable insights. In certain studies, we further delved into the comparison between individuals with ADHD on medication and those with ADHD not on medication, which offered a more nuanced understanding of the relationship. Furthermore, we closely examined the characteristics of the fractures in these studies.

Study Characteristics

We conducted a comprehensive review of nine research papers, involving approximately 357,995 participants assigned to various study groups. Among these finalized studies, four adopted a cohort design, three followed a case-control approach, one combined elements of both cohort and case-control methodologies [22], and one was a descriptive cross-sectional study [16]. All of these studies focused on examining the connection between ADHD and fractures, with one of them specifically investigating the dose-response relationship between MPH and fractures. Some of the studies also considered the impact of MPH on the risk of fractures. The detailed characteristics of the included studies can be found in Table 5, Table 6, and Table 7 of the review.

**Table 5:** Detailed geographic characteristics of the included studies * Cases included children with a first injury occurring during the study period and those who had a weight measurement within a six-month period before and after the injury ** Controls were children who did not experience traumatic injuries during the study period, matched by exact year of period, matched by exact year of birth, and sex at a ratio of 1:1 ADHD, attention deficit hyperactivity disorder

Study/year	Country	Study setting	Study design	Duration	Sample size		Age range	Sex
					ADHD	Control group		Male (in ADHD cohort)
Ziv-Baran et al. [12]	Israel	Meuhedet: electronic data warehouse	Cohort (retrospective)	16.4 years	31,330	62,660 (1:2)	6-18 years	65.5%
Sidrak et al. [21]	United States	TriNetX research database	Cohort (retrospective)	20 years	231,185	354,429	<25 years	65.1%
Alqarni et al. [16]	Saudi Arabia	Three governmental and three private settings in Aseer region, Southwestern Saudi Arabia	Descriptive cross-sectional study	12-month period	163		2-15 years	71.2%
Shem-Tov et al. [22]	Israel	Maccabi’s Healthcare Database	Cohort study	15.5 years	289,21	308,77	12-20 years	65.9%
			Case-control study		8,000 cases*	3,265**		65.3%

**Table 6:** Detailed geographic characteristics of the included studies ADHD, attention deficit hyperactivity disorder

Study/year	Country	Study setting	Study design	Duration	Sample size		Age range	Sex (male)
					Traumatic injuries (or the number of individuals with fractures)	Controls		
Genç et al. [4]	Turkey	Orthopedics and traumatology clinic of Bağcılar Training and Research Hospital	Case-control study	7 months	41	41	3-17 years	65.8%
Karayagmurlu et al. [27]	Turkey	Orthopedics clinic, Gaziantep University Medical Faculty Hospital	Case-control study	10 months	92	60	4-18 years	63.1%
Duramaz et al. [6]	Turkey	Emergency orthopedics outpatient clinic at Bakirkoy Dr. Sadi Konuk Training & Research Hospital, Tevfik Saglam, Istanbul	Case-control study	2 years	138	106	6-16 years	
Prasad et al. [26]	United Kingdom	Clinical Practice Research Datalink (CPRD)	Cohort study	14 years	15,126	263,724	3-17 years	84.6 % for ADHD with fractures

**Table 7:** Details of the outcome characteristics of the included studies *** Patient selection ADHD, attention deficit hyperactivity disorder; AP, anterior to posterior; CDI, Children’s Depression Inventory; CPRS, Conners’ Parent Rating Scale; KSADS-PL, Kiddie Schedule for Affective Disorders and Schizophrenia-Present and Lifetime; MPH, methylphenidate; NOS, not otherwise specified; SCARED, Screen for Child Anxiety Related Disorders; T-DSM-IV-S, Turgay DSM-IV-Based Child and Adolescent Behavior Disorders Screening and Rating Scale-Parents Form

Study/year	Identification method			Outcomes reported	Fracture types reported
	ADHD		Fracture		
Ziv-Baran et al. [12]	ICD-9 codes 314.00-314.01		Coded diagnosis, and classification according to ICD-9 codes 800-829	Fractures	Upper limb (all different groups); lower limb (all different groups); face and skull and trunk (all different groups); other NOS
Sidrak et al. [21]	ICD: F90		Coded diagnosis, classified according to ICD-10	Fractures	Central, upper, and lower; any fractures
Alqarni et al. [16]	Files diagnoses		Files diagnosis	Fractures, injuries, burns, deep injuries, broken or lost teeth, eye injuries, jaw injuries, and anomalies	Overall
Shem-Tov et al. [22]	Cohort study	ICD-9-CM-314.01, 314.00 codes	ICD-9-CM- 800-809, 810-829	Fractures, dislocations, sprains, intracranial injuries, internal injuries of the thorax, abdomen, and pelvis; open wounds, contusions, burns, and injuries to the nerves and spinal cord	Fracture of the skull, neck, and trunk; fracture of the upper or lower limb
	Case-control study	At least one purchase of MPH over the study period***			
Genç et al. [4]	KSADS-PL version		Physical examination and conventional lateral and AP view radiograph images	Fractures	Supracondylar fracture of humerus
Karayagmurlu et al. [27]	CPRS		Patients admitted with traumatic injuries	Fractures, dislocations, soft tissue trauma, and sprains	Upper extremity fractures and lower extremity fractures
Duramez et al. [6]	T-DSM-IV-S, SCARED, and CDI		Patients admitted with extremity fractures	Fractures	Extremity fractures, upper and lower, are then classified into individual extremity bone groups as well
Prasad et al. [26]	ICD-10; at least one diagnosis code or at least one drug code for ADHD		Read codes, for the injuries and ICD-10 or OPCS4 codes	Fractures, thermal injuries, and poisonings	Any fractures; long-bone fractures
Schermann et al. [20]			ICD-9 excluding stress fracture diagnosis (Code M84.3)	Fractures	Fractures

Discussion

The most important findings of the study are discussed below in three categories as follows.

ADHD: Factors and Fracture Characteristics

Every study included in the review demonstrates a higher occurrence of fractures in the ADHD group when compared to the non-ADHD group [4,6,12,16,20-22,26,27]. There is a notably stronger association between ADHD and more serious injuries, such as skull, neck, and trunk fractures, intracranial injuries without a skull fracture, and injuries to the nerves and spinal cord, compared to less severe injuries [20,28]. Hence, there is an increased incidence of hospitalizations due to severe injuries in the ADHD group [6].

In the ADHD and non-ADHD cohorts, the upper limb was the most frequent site of fractures (67.0% vs. 60.1%), followed by the lower limb (18.8% vs. 17.5%), the face or skull (4.3% vs. 4.0%), and the trunk (3.7% in both groups). To be more specific, fractures of the radius and ulna were more prevalent than fractures of any other bones in both groups. The most commonly fractured bone in the lower limb was found to be the tibia [6,12].

Mechanisms and Clinical Implications

There are different scales developed both for screening and determining the severity of symptoms associated with ADHD. A few of them, which were used in recent studies, are mentioned here. These scales offer a systematic approach to identifying, diagnosing, and quantifying the severity of ADHD symptoms. They help clinicians make informed treatment decisions and enable researchers to collect standardized data for a deeper understanding of the disorder.

Karayagmurlu et al. used Conners’ Parent Rating Scale (CPRS), which consists of four subscales: (1) CPRS-inattention (CPRS-IA); (2) CPRS-hyperactivity (CPRS-HA); (3) CPRS-oppositional defiant disorder (CPRS-ODD); and (4) CPRS-conduct disorder (CPRS-OD). They found significantly higher scores for hyperactivity for patients who were getting admitted to orthopedics with complaints of trauma. Similarly, a diagnosis of ADHD has been demonstrated to elevate the likelihood of experiencing a traumatic fracture, likely due to the behavioral traits linked to the condition. These traits encompass inattention, hyperactivity, impulsive actions, lack of coordination, aggression, challenges in motor coordination, an elevated inclination to disregard rules in recreational activities, and a diminished focus on safety measures or engaging in risky behaviors [19,20,26,29-32].

Another study used scales including the Turgay DSM-IV-Based Child and Adolescent Behavior Disorders Screening and Rating Scale-Parents Form (T-DSM-IV-S), the Screen for Child Anxiety-Related Emotional Disorders (SCARED), and the Children’s Depression Inventory (CDI). These forms were filled out by the parents of patients two days after the injury, so as to prevent any impact of an acute emotional response on answers. Their discovery indicated that incidents like slips or trips, pedestrian mishaps, high falls, and bicycle-related fractures occurred more frequently than injuries from playground activities, sports, or altercations. This observation underscores the recurring connection between ADHD-related attributes, such as clumsiness, inattentiveness, disregard for risk, and impulsivity, contributing to accidents [6].

Conversely, injuries occurring in playgrounds, sports, or fights might be attributed to impolite and aggressive conduct, often associated with behavioral disorders, rather than hyperactivity [6].

Additional factors that showed a significant connection with patients who had both ADHD and fractures included being male, having a reported tendency toward injuries according to parents, and having a previous history of trauma [4,6].

Gender disparity is a natural occurrence because neurodevelopmental disorders like ADHD tend to be more prevalent among males [4]. Although fractures were more common in males compared to females, the hazard ratio for fractures was found to be higher in females. The rates might be lower in girls than boys; however, they are higher in the ADHD cohort as compared to the non-ADHD matched cohort [12,33].

Some studies in the past have presented findings showing a relationship between injuries in patients with low socioeconomic status (SES) with and without ADHD. Shem-Tov et al. revealed a 94% higher incidence of injuries in children with low SES with ADHD than low SES without ADHD [22]. The findings in this study indicated that there may be a relationship between SES and the risk of injury (increases with age and is higher among individuals with low SES). Nonetheless, a 2018 study explored the connection between the SES of children and psychiatric factors, yet it did not establish a noteworthy correlation between the two [6].

As we discussed, a previous history of trauma was significantly associated with ADHD patients; hence, in agreement with the same, it was seen that re-fractures are also common in these groups. Duramaz et al. observed a re-fracture rate of approximately 38.4% within their study cohort. These re-fractures were most frequent in the vicinity of the wrist, particularly involving the distal radius and forearm shaft. The primary contributors to these re-fractures were activities marked by recklessness and danger, along with depressive moods and unmanageable behaviors [6]. Other studies also supported the evidence; they found a significantly higher risk of having multiple fractures in children who are diagnosed with ADHD when compared with non-ADHD matched cohorts. The ratios were as follows for two fractures (HR 1.32, 95% CI 1.26-1.38, p < 0.001) or three fractures (HR 1.35, 95% CI 1.24-1.46, p < 0.001) [12].

Karayagmurlu et al. also found higher subscale scores on CPRS-IA and CPRS-HA in patients with recurrent trauma. It might be explained as ADHD is a chronic neurobehavioral condition; hence, it is present in the majority of an individual’s life, thus increasing the probability of presenting with recurrent traumas [27,34].

MPH and Fracture Risk

The median age for starting treatment was around 8.24 years, and this was consistent for both boys and girls [12].

Studies have been conducted to gain insights into the risks associated with MPH. In all studies investigating the impact of MPH, it is consistently observed that the overall fracture risk diminishes with the utilization of these medications. However, we might see a slight change in responses when different characteristics of cohorts are compared [12,20,21].

MPH decreases the risk of various types of fractures (overall, central, and upper limb). However, it might not have any significant difference in reducing the risk of lower limb fractures [21].

Studies have been done that involve comparing a cohort of ADHD patients on medication with two other cohorts: those with ADHD but not on medication and patients without ADHD (who are carefully matched for all other relevant variables). It depicts that treatment in the ADHD cohort reduces the risk of experiencing a fracture, although it might remain slightly elevated when compared to the group without ADHD. However, the use of medication significantly reduces the risk of fracture among children with ADHD (p < 0.001) when compared among themselves [12]. To understand and compare the responses in these three different cohorts, it will be useful to dive further into them. Various aspects of MPH therapy exhibit distinct impacts on its effectiveness, including factors such as the type of medication (short-acting, intermediate-acting, and long-acting), dosage intensity (low, medium, and high), and duration of treatment. Medium- and long-acting are associated with significantly decreased risk. On the other hand, short-acting was not able to reduce the risk significantly. In regards to the proportion of days covered (PDC), there was a decreased trend in all the PDC groups, with significantly reduced risk in the low PDC group (low PDC <0.32, with 270 days being complete coverage of yearly weekdays) [22].

In regards to dose, there are variable results available, especially when we consider higher dosages [20,22]. Research conducted in Israel reveals an inverse dose-response relationship, particularly among males, indicating a negative effect of MPH usage on the risk of fractures. Even in females, despite the absence of a dose-response effect, it was evident that using MPH led to a reduced risk of fractures [20]. It has been seen that the negative dose-response effect in males was persistent, meaning the highest exposure group depicted the lowest risk [20].

Then there is a claim that MPH has a negative effect on bone mineral density (BMD) [35,36]. Hence, there have been many discussions on the use of MPH for reducing fractures. Stimulant medications like MPH increase the levels of catecholamines (dopamine and norepinephrine) at synapse levels by inhibiting their reuptake. Physiologically, during bone remodeling, sympathetic neurons in bones release catecholamines like norepinephrine, which, in turn, inhibits bone formation [37,38] and increases the resorption of bones [39,40]. This sympathetic innervation and bone remodeling are mediated by beta-2 adrenergic receptors expressed on the surface of osteoblasts [41,42].

Another mechanism is by altering the levels of leptins. Leptin promotes the activity of osteoblasts [43] and downgrades the differentiation and proliferation of osteoclasts [44]. However, stimulants lead to decreased levels of leptins. Hence, long-term use of these medications decreases bone health.

It has been shown through recent studies that males with ADHD who were prescribed these stimulant medications, especially for more than three months, have reduced bone mineral content and BMD for the total body, especially at the lumbar spine and pelvis (femur), when compared with patients who were not prescribed these drugs [35,45,46].

In spite of all the above side effects, several studies have consistently proven that MPH treatment in patients with ADHD is associated with a reduced risk of fracture. This effect of MPH is linked to the behavioral modification effect of the stimulant class of drugs [12,20,22].

Considering the elevated prevalence of ADHD in children, we recommend employing various ADHD symptom checklists. Additionally, it is advisable to inquire with parents about their child’s hyperactive or impulsive behaviors, any prior incidents of injury, and their child’s tendency toward accidents. Seeking a neurodevelopmental and behavioral evaluation when there are suspicions of ADHD may enable early diagnosis and intervention. This proactive approach can help prevent additional injuries and potential comorbid conditions, ultimately reducing overall treatment costs.

Hence, there is an immediate need to formulate additional strategies or guidelines for preventing fractures in children with ADHD. While the causal relationship has been elucidated, its real value lies in the development and implementation of practical strategies in the daily lives of children with ADHD, encompassing settings such as schools, homes, and playgrounds.

Limitations

Our paper is not exempt from limitations. It lacks randomized clinical trials, which are considered a robust form of evidence. The review relies solely on qualitative evidence, and no quantitative analysis has been conducted. A meta-analysis would offer deeper insights into the association between ADHD, MPH, and fractures, allowing for a more precise assessment of their significance. Furthermore, the impact of other potential variables, such as mental health conditions, has not been investigated. Additionally, this review does not encompass emerging medications that are gaining prominence in the market.

## Conclusions

ADHD significantly increases fracture risk, while MPH plays a role in mitigating this risk by addressing ADHD-related mechanisms and affecting bone cells. On the basis of the relationship evident in our review, early ADHD detection can lead to timely interventions, including nonpharmacological methods like parent education and behavioral interventions tailored to developmental stages. Orthopedic surgeons should prioritize ADHD recognition through screening tools and direct inquiries, reducing injury risk through proactive measures and facilitating early identification and treatment of ADHD. Healthcare staff should educate children, adolescents, and caregivers on injury prevention during various stages of diagnosis and follow-up. Targeted screening efforts are required for the groups at high risk of ADHD, such as boys, children with a history of fractures, or those who are deemed accident prone.
